# Extended coagulation profile of children with Long Covid: a prospective study

**DOI:** 10.1038/s41598-022-23168-y

**Published:** 2022-11-01

**Authors:** Leonardo Di Gennaro, Piero Valentini, Silvia Sorrentino, Maria Antonietta Ferretti, Erica De Candia, Maria Basso, Stefano Lancellotti, Raimondo De Cristofaro, Cristina De Rose, Francesco Mariani, Rosa Morello, Ilaria Lazzareschi, Louise Sigfrid, Daniel Munblit, Danilo Buonsenso

**Affiliations:** 1 Department of Diagnostic Imaging, Radiotherapy, Oncology and Haematology, Hemorrhagic and Thrombotic Diseases Center, Fondazione Policlinico Universitario A. Gemelli IRCCS, Rome, Italy; 2 Department of Woman and Child Health and Public Health, Fondazione Policlinico Universitario A. Gemelli IRCCS, Rome, Italy; 3 Istituto di Ematologia, Università Cattolica del Sacro Cuore, Rome, Italy; 4 Nuffield Department of Medicine, International Severe Acute Respiratory and Emerging Infection Consortium Global Support Centre, Centre for Tropical Medicine and Global Health, University of Oxford, Oxford, UK; 5 Department of Paediatrics and Paediatric Infectious Diseases, Institute of Child’s Health, Sechenov First Moscow State Medical University, Moscow, Russia; 6 Inflammation, Repair and Development Section, Faculty of Medicine, National Heart and Lung Institute, Imperial College London, London, W2 1PG UK; 7 Centro di Salute Globale, Università Cattolica del Sacro Cuore, Rome, Italy

**Keywords:** Medical research, Pathogenesis

## Abstract

Emerging data suggests that endotheliopathy changes can be associated with post covid condition (PCC) in adults. Research on the matter in children is lacking. We analyzed an extended coagulation profile including biomarkers of endothelial damage in children with PCC and compared it with a control group of children that fully recovered post- SARS-CoV-2 infection. A case-control study enrolling children below 18 years of age with previous microbiologically confirmed SARS-CoV-2 infection in a pediatric post-covid unit in Italy ≥ 8 weeks after the initial infection. Samples were taken at 8 and 12 weeks after the SARS-CoV-2 diagnosis and analyzed for coagulation profiling (fibrinogen, prothrombin time, international normalized ratio, activated partial thromboplastin time, d-dimers, factor VIII coagulant activity, plasma von Willebrand factor (VWF) antigen and VWF ristocetin cofactor (RC)). We compared coagulation profiles in samples from children identified with PCC (at least one, or three or more symptoms, which could not be explained by an alternative diagnosis, at the 8- and 12-week follow-up assessment using the pediatric Long Covid International Severe Acute Respiratory and Emerging Infection Consortium (ISARIC) survey. Seventy-five children were enrolled, 49.3% were females, the median age was 10.2 (IQR 4.9) years. Forty-six (61%) of the children had at least one persisting symptom at the eight weeks post-onset, (PCC8); 39/75 (52%) had persistent symptoms for more than 12 weeks (PCC12) and 15/75(32%) had at least three persisting symptoms (PCC ≥ 3) at 12 weeks. Children with PCC presented more frequently with abnormal D-Dimer levels above the reference range compared to children that had fully recovered at the 8–12 weeks (39.1% vs. 17.2%, *p* = 0.04), and 12 week follow up or more (41% vs. 17.2%, *p* = 0.05), and in children with three or more symptoms at 12 weeks follow up compared to those that had recovered (64.3% vs. 22.2%, *p* = 0.002). For the other coagulation profiles, there were abnormal values detected for VWF, FVIII, RC and Fibrinogen but no significant differences between children with PCC compared to controls. Although the majority of children in our cohort showed coagulation profile within or close to normal ranges, we found that a higher proportion of children with PCC, and specifically those with a more severe spectrum characterized with three or more persisting symptoms, had abnormal D-dimer levels compared to other children that fully recovered from an acute SARS-CoV-2 infection.

## Introduction

After more than two years of the pandemic, it is increasingly evident that the outcomes of SARS-CoV-2 infection extend far beyond recovery from acute illness or death^1^. Several independent follow-up studies of adults affected by COVID-19, with or without in need of hospitalization during the acute phase, have shown that an estimated 15–50% of patients still experience a wide range of symptoms and complications, affecting their physical and psychological wellbeing months after the initial diagnosis^2,3^. Recent studies have also identified organ damage in the lungs, cardiovascular system, kidney and brain of COVID-19 survivors^1–4^. This complex cohort of sequelae persisting after COVID-19 and without an alternative diagnosis is commonly referred to as Long Covid or Post-Covid Condition (PCC)^5^.

Although the etiology of PCC is still unknown, recent studies are providing a growing increasing number of evidence that factors like viral persistence, chronic subtle inflammatory events or immune dysregulation, autoimmunity, reactivation of latent infections, deconditioning, mental factors and chronic endothelial dysfunction may all play a role^6^. Specifically, there is particular focus on chronic endotheliopathy and circulating microclots as relevant contributing factors^7,8^, that might explain several of the key symptoms documented such as chest pain, musculoskeletal pain, pulmonary hypoperfusion) and the well-established cardiovascular risk following SARS-CoV-2 infection. In addition, other readily available and routinely used parameters like D-Dimers, von Willebrand factors and FVIII and other routine coagulation parameters have also been used as markers of endothelial activation and also assessed in adults with PCC, showing that Willebrand factor antigen (VWF:Ag), VWF propeptide (VWFpp), and factor VIII were significantly elevated in convalescent COVID-19 compared with control^9^.

While all these mechanisms have been extensively studied in adults with PCC, there are no studies to date in the pediatric population. There is, an increasing recognition of that children can also develop PCC, in some with profound impact on education and activities^10–14^. However available evidence is (1) mostly based on online and retrospective studies, (2) without control groups, or with inappropriate controls, or (3) patients were identified based on a single negative PCR test or the absence of IgG anti-SAR-CoV-2, neither of which are valid tests^15–27^. For these reasons, most of the current debate on pediatric PCC has focused on if PCC is a real pathologic event in children caused by SARS-CoV2 infection, or an indirect consequence due to the restrictive measures during the pandemic^10–27^. This has severely hampered the progress and the development and implementation of studies focusing on diagnostics and biomarkers, as well as trials into treatments.

Nevertheless, given the growing number of children presenting with persisting symptoms after SARS-CoV-2 infection in our institution in Rome in Italy, and reported internationally, we set up a study to explore pathophysiological mechanisms and biomarkers in children with PCC. In response to the emerging evidence on endotheliopathy as potential mechanism behind some of the sequalae identified in adults with PCC, we performed similar studies in children. The aim of this case control study is to analyze an extended coagulation profile, including biomarkers of endothelial damage in children with PCC, compared with a control group of children that fully recovered after SARS-CoV-2 infection.

To our knowledge, this is the first study assessing coagulation profiles in children with PCC compared to controls.

## Methods

### Study population

This is a prospective study of children younger than 18 years of age with a previous microbiologically confirmed diagnosis (based on SARS-CoV-2 detected on nasopharyngeal swab by RT-PCR) of SARS-CoV-2 infection that were assessed in our pediatric post-covid outpatient clinic in Rome, Italy. In our outpatient clinic, we evaluated children that had fully recovered from acute infection and those that presented with persisting symptoms. Children can be sent to the post-covid unit either after discharge from our Institution, or directly sent from the family pediatricians (and therefore not seen at baseline during acute infection). We developed a protocol to assess children with PCC, which has been described previously (https://isaric.org/research/covid-19-clinical-research-resources/paediatric-follow-up/, full version included in the supplementary material). The assessments took part from October 1st 2021 to March 31st 2022.

Therefore, the following categories of children were enrolled at the first outpatient visit if fulfilling study criteria:

*PCC8 group* Children with persisting symptoms for at least 8 weeks after SARS-CoV-2 infection, that cannot be explained by an alternative diagnosis **(**PCC8 group). In lack of a case definition for PCC when we started the study, we initially defined children with PCC as those experiencing at least one persisting symptom for more than eight weeks after the initial SARS-CoV-2 diagnosis.

*PCC12 group* During the study period WHO released a definition of PCC in adults, which defined PCC as at least one symptom persisting for 12 weeks or more^5^. Although at the time of beginning of this study there was not yet a definition for children, we created a subgroup of children fulfilling the adult definition (PCC12), which is in line with a later definition released the pediatric Delphi consensus of the CLOCK study^28^.

*PCC* ≥ *3 group* Since the diagnosis of PCC is based on symptoms that may be non-specific, we also created a further subgroup of children who were experiencing at least three persisting symptoms, in the hypothesis that this might provide further specificity for the definition of PCC. Symptoms had to persisting for at least 8 weeks, and we created a further subgroup of children with at least three persisting symptoms lasting at least 12 weeks (subanalyses available in the supplementary methods).

In all PCC definitions, persisting signs/symptoms could not be explained by other known conditions AND had a clear negative impact on daily functioning (further details in the supplementary material).

### Controls

*Fully recovered children* This group included those that reported no persisting symptoms after acute SARS-CoV-2 infection at time of follow-up post- onset of acute COVID-19 symptoms (at least 8 weeks).

Disease severity during acute infection was classified as asymptomatic, mild, moderate, severe, according to the adapted classification by Buonsenso et al.^29^.

#### Inclusion and exclusion criteria

The following inclusion criteria were used:

- Children aged 0–18 years
- The child presented in a primary or secondary care medical facility due to COVID-19 illness.
- Laboratory (RT-PCR) diagnosis of acute SARS-CoV-2 infection
- Assessed at least 8 weeks days after the first positive test for SARS-CoV-2 PCR
- Parent’s/caregiver’s/guardian’s consent to participate.

Exclusion criteria:

Children with

- Suspected PCC children but eventually diagnosed with celiac diseases, anemia, autoimmune diseases, hypothyroidisms, diabetes, hepatitis, blood malignancies, as per our protocol^6^. During our first assessment, in case of persisting symptoms, we perform a number of blood tests in addition to coagulation profile, including routine blood tests, celiac disease, autoimmunity, etc., which allow to make alternative diagnosis in a short time and exclude patients from the enrollment as a PCC child.
- Children with known pre-existing mental health issues that made difficult to understand if Covid-19 might had an impact on daily functioning or it was due to the pre-existing condition
- Confirmed or suspected primary or acquired immune compromising conditions, recent or current administration of immune suppressive therapies, or other diseases affecting the immune system, or known coagulation disorder or any ongoing treatment with anticoagulants/antiaggregants, or pre-existing asthma.
- Fulfilling WHO’s criteria for MIS-C were excluded, since recent studies suggest a specific immunological signature for this condition, and abnormalities of coagulation biomarkers are well-established^30,31^.
- Recovered children that had Covid-19 less than 8 weeks before.

### Coagulation studies

To identify abnormalities of the coagulation system in these patients, routine hemostatic markers were monitored including fibrinogen, prothrombin time (PT), international normalized ratio (INR), activated partial thromboplastin time (aPTT), d-dimers, factor VIII coagulant activity, plasma von Willebrand factor (VWF) antigen and VWF ristocetin cofactor (RC) activity. Blood samples were drawn within 6 h of hospital evaluation by the evaluating researcher and analysis done immediately in a specialized laboratory for coagulative disorders in our Institution.

The ACL 700 Top analyzer (Werfen Group, Milano, Italy), a fully automated random access analyzer for coagulometric, chromogenic and immunologic measurements was for PT, INR, fibrinogen level, aPTT and D-dimer analysis.

Plasma VWF antigen and VWF ristocetin cofactor activity were measured using an automatic chemiluminescent test (HemosIL AcuStar VWF; Instrumentation Laboratory, Werfen Group, Milano, Italy). FVIII coagulant activity was measured with a chromogenic assay (Chromogenix Coatest FVIII kit, Instrumentation Laboratory, Bedford, Massachusetts, USA) using the same ACL Top 700 instrument.

The anticoagulant vial of choice for coagulation studies is 3.2% Sodium Citrate (Blue Top Tube).

All experiments were performed in accordance with the relevant guidelines and regulations.

### Statistical analysis

Given the lack of evidence in the literature, this is configured as a pilot study, using convenience sampling. As such, no formal sample size calculation was needed, and inclusion based on standards for pilot studies, recommending a minimum sample size of 20 subjects for each group^32^.

For continuous variables the Kolmogorov–Smirnov test was used to assess whether the distribution was normal or not. Categorical variables were reported as count and percentage. All the continuous variables presented a non-normal distribution and are therefore expressed as median and interquartile range (IQR 25–75%). Statistical comparisons between two groups were obtained by Chi-squared tests or Fisher’s exact tests for categorical variables and Mann–Whitney U-test for continuous variables if not normally distributed. *P* value < 0.05 was considered statistically significant. The Kruskal–Wallis test was performed to compare the number of symptoms at follow-up between patients with asymptomatic, mild and moderate acute SARS-Cov2 infection.

To investigate the role of potential risk factors of developing persistence of symptoms we calculated odds ratio and 95% confidence intervals (OR, 95% CI), from a 2 × 2 table, for the symptoms of the acute phase that resulted statistically significative associated with the persistence of symptoms at follow-up (muscle pain and articular pain).

All analyses were performed comparing the recovered children with each of the following categories of children with persisting symptoms: PCC8 children, PCC12 children, PCC ≥ 3 children.

Statistical analysis was performed using IBM SPSS Statistics 23.0 software (IBM Corporation, Armonk, NY, USA).

### Ethic committee approval

The study was approved by the ethic committee of the Fondazione Policlinico Universitario A. Gemelli IRCCS, Rome, Italy (ID 3777, prot 0004150/21). Written informed consent was obtained from all participants or legal guardians.

## Results

### Study population characteristics

Of 83 children initially assessed, two with persisting symptoms were excluded because an alternative diagnosis of celiac disease was performed, and six children that fully recovered from initial infection did not agree to participate to the study. Therefore, we enrolled 75 children, 38 males (50.7%) and 37 females (49.3%), the median age was 10,2 years^4,9^ (Table 1). The median follow up time from the time of acute SARS-CoV-2 infection to the visit was 3.3 months (IQR 4.6). Regarding the characteristics of acute SARS-CoV-2 infection, four (5.3%) children were asymptomatic, 60 (80%) had mild disease, 11 (14.7%) a moderate/severe form. Seven children (9.3%) needed hospitalization in the acute phase, and two of then required pediatric intensive care.

**Table 1:** Differences in demographics and clinical characteristics during the acute SARS-CoV-2 infection between patients with persistent symptoms at follow-up and patients recovered.

	Study population (n = 75)	Patients recovered (n = 29)	Patients with persistence of symptoms (PCC children) (n = 46)	p
Gender, n (%) Female	37 (49.3%)	13 (44.8%)	24 (52.2%)	0.8
Age (y), median IQR	10.2 (4.9)	10.1 (3)	10.5 (6.7)	0.34
Follow-up (months), median IQR	3.3 (4.6)	3.1 (6)	3.6 (4.4)	0.9
Comorbidities, n (%)	6 (8%)	2 (6.9%)	4 (8.7%)	1
**Disease severity, n (%)**				
Asymptomatic	4 (5.3%)	2 (6.9%)	2 (4.3%)	0.3
Mild	60 (80%)	25 (86.2%)	35 (76.1%)	
Moderate/severe	11 (14.7%)	2 (6.9%)	9 (19.6%)	
Hospitalization, n (%)	7 (9.3%)	2 (6.9%)	5 (10.9%)	0.7
PICU hospitalization, n (%)	2 (2.7%)	1 (3.4%)	1 (2.2%)	1
Number of symptoms, n (%) median IQR	3 (3)	2 (2)	4 (4)	0.005
Fever, n (%)	52 (69.3%)	17 (58.6%)	35 (76%)	0.1
Days of fever, median IQR	2 (3)	1 (2)	2 (3)	0.002
Rhinitis, n (%)	27 (36%)	12 (41.4%)	15 (32.6%)	0.4
Anosmia, n (%)	22 (29.3%)	5 (17.2%)	17 (37%)	0.06
Dysgeusia, n (%)	18 (24%)	5 (17.2%)	13 (28.3%)	0.2
Cough, n (%)	27 (36%)	8 (27.6%)	19 (41.3%)	0.22
Dyspnea at rest, n (%)	4 (5.3%)	1 (3.4%)	3 (6.5%)	1
Dyspnea under exertion, n (%)	5 (6.7%)	1 (3.4%)	4 (8.7%)	0.6
Asthma, n (%)	2 (2.7%)	0 (0%)	2 (4.3%)	0.5
Chest pain, n (%)	7 (9.3%)	1 (3.4%)	6 (13%)	0.23
Joint pain, n (%)	12 (16%)	1 (3.4%)	11 (23.9%)	0.02
Muscle pain, n (%)	19 (25.3%)	2 (6.9%)	17 (37%)	0.005
Asthenia, n (%)	28 (37.3%)	7 (24.1%)	21 (45.7%)	0.06
Headache, n (%)	31 (41.3%)	10 (34.5%)	21 (45.7%)	0.33
GI disorders, n (%)	10 (13.3%)	1 (3.4%)	9 (19.6%)	0.07
Rash, n (%)	6 (8%)	1 (3.4%)	5 (10.9%)	0.39

*ICU* intensive care unit, *GI* gastrointestinal.

**Figure 1:**
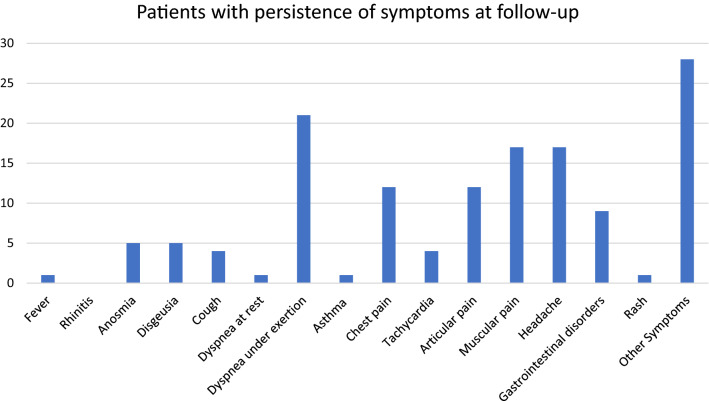
Persisting symptoms reported at 8 weeks or more follow-up evaluation, using the ISARIC assessment tool (supplementary materials). Y-axis refers to numbers.

Of the 75 enrolled children, 46 (61.3%) experienced at least one unexplained, persisting symptom at the 8-weeks follow-up (PCC8), 39/68 (57.3%) still had symptoms after 12-weeks (PCC12). The symptoms are presented in Fig. 1. Dyspnea during and after mild activities (post-exertional malaise), musculoskeletal pain, asthenia/fatigue and neuropsychiatric issues were the most frequently reported. The group of children that developed PCC8 had, a statistically significant higher number of symptoms (2 (1.5) vs. 4 (4.2) *p* = 0,005), more days of fever (1 (2) vs. 2 (3.25) *p* = 0.002), and more frequently presented joint pain (23% vs. 3.4%; *p* = 0.02) and muscle pain (37% vs. 6.9%; *p* = 0.005) during the acute SARS-CoV-2 infection, (Table 1). Similar results were obtained when only children with ≥ 3 persisting symptoms were considered as PCC (PCC ≥ 3, Table S1), or when only children with persisting symptoms for ≥ 12 weeks were considered as having PCC (PCC12, Table S2), or when both the persistence of at least three symptoms (PCC ≥ 3) for at least 12 weeks (PCC12) were considered as criteria of PCC (Table S3).

### Coagulation profiles

Details of the extended coagulation profile at the follow-up of the two cohorts of children are reported in Table 2. Overall, both groups of patients presented abnormal coagulation profiles at follow-up.

**Table 2:** Differences in the coagulation profile between patients with persistence of symptoms and patients recovered at follow-up.

	Normal values	Patients recovered (n = 29)	Patients with persistence of symptoms (PCC children) (n = 46)	p
VWFAg, median IQR	70–140%	91 (21.5)	87 (33.4)	0.66
Abnormal VWFAg, n (%)		5 (17.2%)	13 (28.2%)	0.27
RC, median IQR	70–140%	90 (27.5)	86 (27)	0.65
Abnormal RC, n (%)		4 (13.8%)	9 (19.6%)	0.75
F VIII, median IQR	70–140%	93 (35)	90.5 (24)	0.84
Abnormal F VIII, n (%)		4 (13.8%)	11 (23.9%)	0.38
PT, median IQR	0.9–12 s	11 (0.3)	11.1 (0.4)	0.06
Abnormal PT, n (%)		0 (0%)	3 (6.5%)	0.27
aPTT, median IQR	24–37 s	32 (8.2)	34 (6.2)	0.12
Abnormal aPTT, n (%)		7 (24.1%)	12 (26%)	0.85
INR, median IQR	0.9–1.2	1.1 (0.2)	1.1 (0.1)	0.33
Abnormal INR, n (%)		0	0	
Fibrinogen, median IQR	200–400 mg/dl	214 (112)	262 (104)	0.3
Abnormal fibrinogen, n (%)		12 (41.4%)	13 (28.2%)	0.24
D-dimer, median IQR	< 500 ng/ml	211 (200)	311 (426)	0.06
Abnormal d-dimer, n (%)		5 (17.2%)	18 (39.1%)	0.04

*VWFAg* von willebrand factor antigen, *RC* ristocetin cofactor, *F VIII* factor VIII, *PT* prothrombin time, *aPTT* activated partial thromboplastin time.

PCC8 children presented more frequently abnormal PT, VWF, PT, aPTT and D-Dimer values, although differences were statistically significant only for D-Dimers (abnormal D-Dimer values in 39.1 PCC and 17.2% recovered children, *p* = 0.04). Similar results were obtained when we used as PCC definition the presence of at least three persisting symptoms (PCC ≥ 3, Table S4), although in this case both D-Dimer median values (268 ng/ml vs. 590 ng/ml, *p* 0.02) and abnormal levels (23.3% vs. 60% of the respective cohorts, *p* 0.006) were significantly higher in PCC ≥ 3 children. Similar differences were obtained when only the persistence of symptoms for at least 12 weeks was considered as an inclusion criteria for PCC (PCC12, Table 3), or when both the persistence of at least three symptoms (PCC ≥ 3) for at least 12 weeks (PCC1) were considered as criteria of PCC/ (Table 4).

**Table 3:** Differences in the coagulation profile between patients with persisting symptoms at 12 weeks or more follow-up (PCC12) and patients recovered.

	Normal values	Patients recovered (n = 29)	Patients with PCC12 (n = 39)	*p*
VWFAg, median IQR	70–140%	91 (21.5)	87 (34.5)	0,4
Abnormal VWFAg, n (%)		5 (17.2%)	12 (30.8%)	0,2
RC, median IQR	70–140%	90 (27.5)	85 (30)	0,5
Abnormal RC, n (%)		4 (13.8%)	8 (20.5%)	0,54
F VIII, median IQR	70–140%	93 (35)	87 (24)	0,69
Abnormal F VIII, n (%)		4 (13.8%)	10 (25.6%)	0,36
PT, median IQR	0.9–12 s	11 (0.3)	11.1 (0.4)	0,07
Abnormal PT, n (%)		0 (0%)	3 (7.7%)	0,25
aPTT, median IQR	24–37 s	32 (8.2)	34 (6.9)	0,15
Abnormal aPTT, n (%)		7 (24.1%)	11 (28.2%)	0,7
INR, median IQR	0.9–1.2	1.1 (0.2)	1.1 (0.1)	0,33
Abnormal INR, n (%)		0	0	
Fibrinogen, median IQR	200–400 mg/dl	214 (112)	289 (106)	0,14
Abnormal fibrinogen, n (%)		12 (41.4%)	11 (28.2%)	0,26
D-dimer, median IQR	< 500 ng/ml	211 (200)	311 (418)	0,07
Abnormal d-dimer, n (%)		5 (17.2%)	16 (41%)	0,04

Only patients assessed after 12 weeks were included in these comparisons.

*VWFAg* von willebrand factor antigen, *RC*ristocetin cofactor, *F VIII* factor VIII, *PT* prothrombin time, *aPTT* activated partial thromboplastin time.

**Table 4:** Analysis performed excluding patients with 8–11 weeks of follow-up; the table shows the differences in the coagulation profile between patients with persistence of 3 or more symptoms at ≥ 12 weeks follow-up and patients with persistence of less than 3 symptoms at ≥ 12 follow-up.

	Normal values	Patients without persistence of more than 3 symptoms (PCC ≥ 3) and follow-up of at least 12 weeks (n = 54)	Patients with persistence of more than 3 symptoms (PCC ≥ 3) and follow-up of at least 12 weeks (n = 14)	*p*
VWFAg, median IQR		87 (23.3)	89 (40.9)	0.3
Abnormal VWFAg, n (%)	70–140%	13 (24.1%)	4 (28.6%)	0.74
RC, median IQR		85 (24.8)	92.5 (34.8)	0.58
Abnormal RC, n (%)	70–140%	9 (16.7%)	3 (21.4%)	0.7
F VIII, median IQR		89.5 (29)	90.5 (35)	0.6
Abnormal F VIII, n (%)	70–140%	12 (22.2%)	2 (14.3%)	0.72
PT, median IQR		11 (0.3)	11.1 (0.5)	0.18
Abnormal PT, n (%)	0.9–12 s	1 (1.9%)	2 (14.3%)	0.1
aPTT, median IQR		32.7 (8.5)	34 (6)	0.6
Abnormal aPTT, n (%)	24–37 s	15 (27.8%)	3 (21.4%)	0.75
INR, median IQR		1.1 (0.2)	1.1 (0.1)	0.31
Abnormal INR, n (%)	0.9–1.2	0	0	
Fibrinogen, median IQR		245 (113)	304.5 (50)	0.1
Abnormal fibrinogen, n (%)	200–400 mg/dl	20 (37%)	3 (21.4%)	0.35
D-dimer, median IQR		268 (236)	595 (435)	0.01
Abnormal d-dimer, n (%)	< 500 ng/ml	12 (22.2%)	9 (64.3%)	0.002

Only patients assessed after 12 weeks were included in these comparisons.

*VWFAg* von willebrand factor antigen, *RC* ristocetin cofactor, *F VIII* factor VIII, *PT* prothrombin time, *aPTT* activated partial thromboplastin time.

We evaluated whether there were statistically significant differences between the values of the coagulation parameters collected at follow-up and different severities of acute COVID. The only statistically significant result was that relating to FVIII, higher in patients with moderate/severe forms of acute COVID than in those with mild and asymptomatic forms (*p* = 0,03).

In Table 5 are reported the risk factors for developing PCC. The presence of joint pain (OR 8.8, CI 1–72.3) and the presence of muscle pain (OR 7.9 CI 1.6–37.5), both during the acute phase of infection, are significant risk factors for the persistence of symptoms at follow-up.

**Table 5:** Risk factors for development of persistence of symptoms at follow-up.

	OR (CI)	*P*
Articular pain	8.8 (1–72.3)	0.04
Muscle pain	7.9 (1.6–37.5)	0.009

## Discussion

Available research on Pediatric PCC have mostly focused on observational studies aimed at characterize the burden of PCC in children and the main problems reported by patients or caregivers^10–27^, while only a few case reports or small case series have been done to detect lung or brain functional problems in this cohort of children^12,33,34^. Conversely, research in adult PCC has moved faster, providing evidence that adults with PCC have immunological abnormalities, EBV reactivation, cardiovascular events and, more recently, abnormal endothelial/coagulation events, including circulating microclots.

In this study, we prospectively evaluated an extended coagulation profile in a cohort of children that either recovered or developed PCC after the initial SARS-CoV-2 infection. To our knowledge, this is the first attempt in defining coagulation biomarkers in children with PCC. Investigating the possible relationship between persistence of symptoms and laboratory alterations in coagulative aspects in children can be particularly relevant, since COVID-related morbidity and mortality is largely associated with hypercoagulability and increased risk of venous thromboembolism, leading to thrombo-inflammation in severe condition in adults^35^, but also in children according to a recent study^30^. The pathological features in thromboinflammation share common interacting processes, such as thrombus formation through activation of platelets, endothelial damage, and coagulation cascade and activation of the innate and adaptive immune systems. Thromboinflammation in COVID-19 causes endothelial damage by producing proinflammatory cytokines and activating platelets and the complement system. Moreover, high plasma levels of D-dimer were observed in COVID-19 patients, also after healing, and high D-dimer levels were correlated with a more severe disease course.

In our pediatric cohort, we found that while all median values fell within the normal range, PCC8 children had higher levels of fibrinogen, factor VIII, and Von Willebrand Factor (VWF), though these results were not statistically significant compared with recovered children. PCC8 children presented significantly higher D-Dimer median values though again these values were within the normal range. They also have more frequently abnormal values of D-dimers than the recovered controls. Interestingly, differences remained significant for each sub analysis with the use of different PCC definitions (8- or 12-weeks follow-up, having at least 3 persisting symptoms, or both). Differences between groups were more pronounced when we used a PCC definition of having at least three persisting symptoms, suggesting that this can be a possibly more specific definition of PCC, or that D-Dimers may be more useful to identify a more severe spectrum of pediatric PCC. Interestingly, patients with more than three symptoms and in the combined PCC ≥ 3 and PCC12 definition, those with PCC tended to be older, suggesting that older children may be more physiologically similar to adults in terms of inflammatory/coagulatory responses after SARS-CoV-2 infection.

D-dimer represents the activation of coagulation and fibrinolysis systems^36^ and plays a mechanistic role in thrombo-inflammation in COVID-19^37^.

The coagulation system can be a host defense response against the invasion of infectious agents such as viruses^38^. This important defensive response of the immune system by causing a clot is cause of elevated D-dimer levels following an infectious agent^39^.

In a recent study, increased D‐dimer levels (> 500 ng/ml) were observed in 25.3% patients up to 4 months in adults post‐SARS‐CoV‐2 infection. In contrast, other coagulation (prothrombin time, activated partial thromboplastin time, fibrinogen, platelet count) and inflammation (C‐reactive protein, interleukin‐6, and sCD25) markers had returned to normal in > 90% of convalescent adult patients^40^.

In another study, 15% of the patients recovered from COVID-19, persistent D-dimer elevation was observed after a median of 3 months following COVID-19^30^. Buonsenso et al. showed that D-dimer values play an important role in predicting the more severe spectrum of the SARS-CoV-2 infection in 316 children. In this study D-dimers proved to be the only statistically significant independent risk factor for pediatric intensive care unit admission (OR 1.9, 95% CI 1.11–3.25), highlighting how this parameter can be more useful than all the other parameters detected at the diagnosis in the risk assessment^41^. Also in a study conducted in Wuhan, China, the D-dimer levels were higher among children with acute and severe COVID infection (Univariate OR 17.4)^42^.

D-dimer levels were also found to be risk factor for pulmonary dysfunction among adult survivors of COVID-19 at three-month post-hospital discharge^43^. In our study there was no clinical evidence of macro-thrombosis, although the investigations that can be performed in asymptomatic children are limited due to ethical problem. In fact, so far, D-Dimer level is an important predictor for thromboembolic events in the long-term follow-up, but suggest CT pulmonary angiogram particularly in those who are still symptomatic. Earlier studies have already reported the importance of the D-Dimer as a predictor for thromboembolic events not only in patients with infectious diseases but also for example for recurrent thrombosis after withdrawal of anticoagulation therapy^44,45^.

However, micro-thrombosis cannot be ruled out, which could also account for the symptoms in some hospitalised young patients (9% of cases). However, it is interesting to highlight that the abnormal D-Dimer levels found in our cohort is somehow in line with adults studies showing circulating microclots in adults^7,8^, or with two independent pediatric small case series showing pathological lung perfusion defects in children with PCC, in absence of macroembolic events^12,46^. Therefore, our findings, along with available literature, support the possibility that chronic endothelial inflammation may play a role also in pediatric PCC and reinforce the need of performing further and deeper studies to better investigate new biomarkers of endothelial/platelet hyperactivation, hyperreactivity or chronic inflammation. It is important to highlight, however, that the mechanisms leading pathological lung perfusion defects in children with PCC^12,24^ are not fully elucidated, and not necessarily D-dimers and other coagulation studies may be biomarker of this localized pulmonary events, as other more sensitive biomarkers not available in routine may be more appropriate to detect minor peripheral endothelial event. For example, after our first description of abnormal lung perfusion in an adolescent with PCC^12^, we have performed this test in other 11 children, having in total 4 pathological SPECT, and only one of them had abnormal D-Dimer levels. In fact, it is important to highlight that increased levels of a vascular related pro-inflammatory marker such as coagulative Factor VIII and D-dimer found in a subgroup of patients most probably can only partly explain the pathophysiology of PCC, particularly and perhaps the inflammation-related symptoms such as fatigue, myalgia, joint pain that resulted as major symptoms in our population. Notably, chronic fatigue is frequently a complex syndrome that may have other causes besides inflammation, such as inadequate cerebral perfusion and autonomic nervous system dysfunction, which may also be involved in PCC. Also, it is possible that some of the patients with normal laboratory findings have been misclassified as having PCC while their symptoms could be due to other psychological problems. Since there is no diagnostic test of PCC, this possibility should still be considered.

In the meantime, changes in levels of D-dimer could serve as potential biomarkers of PCC to be used in routine practice, since D-dimers are a routine blood test, easy to perform and available in most settings. As showed in a recent review, there is growing understanding of a possible role of the endothelium in the pathophysiology of PCC^47^ and, although the interpretation of abnormal D-dimer in this context has still to be elucidated, this may suggest the importance of closer follow-up of this subgroup of children or their participation in pharmacological trials, when available in pediatric practice. Importantly, elucidating the biological mechanisms responsible for sustained D‐dimer increases may be of relevance in PCC pathogenesis and has implications for clinical management of these patients.

Our study has some limitations to address. First, the small sample size and single-center design are the major limitations; however, the important preliminary findings of our study reinforce the urgent need of funding larger multicenter studies investigating this topic. Specifically, the control group was small considering that most children fully recover from initial SARS-CoV-2 infection; however, being our center an internationally recognized pediatric PCC center, families with children with persisting symptoms after initial infection are keener to seek medical evaluation at our center, and to participate to clinical studies, compared with those that fully recovered and came back to normal life. This indirect bias selection should be considered in the interpretation of our results, including lack of power to detect statistically significant differences in coagulation studies within the two groups, and also explain the large confidence intervals obtained by our statistical analyses. In fact, it is possible that our study shows a real sign in a possible role for endothelial markers in pediatric PCC, but larger numbers may be needed to find statistically significant differences. Specifically, normal values of such an extensive coagulation profile are poorly studied in children, despite they have peculiar homeostasis. So far, there is not much data on the percentage of the healthy paediatric population with abnormal coagulation parameters at any given time, and probably a control group of thousands of children would be necessary. In fact, most of the data come from studies of adult populations. It is now clear that the physiology of hemostasis in pediatric patients differs widely from that in adults, supporting the hypothesis that children might have natural protective mechanisms that justify such variations. Therefore, the correct interpretation of hemostasis test results in young patients, along with a deep understanding of the normal postnatal development in the human coagulation system, are essential prerequisites to the proper investigation of thrombotic and hemorrhagic problems in pediatric patients. The understanding of physiological age-dependent changes in the coagulation system is crucial to an accurate diagnosis in the case of problems of thrombosis or bleeding, especially in the very young child. In general, young children have decreased physiological levels of coagulation proteins such as factors II, VII, IX, X, XI and XII, and low levels of proteins involved in fibrinolysis (plasminogen and tissue plasminogen activator) and natural coagulation inhibitors (such as antithrombin and protein C and S)^48^. Secondly, baseline coagulation studies during the acute infection are not available. However, such a study in pediatric population would be extremely difficult since most children during acute infection have a mild disease and do not seek medical attention and do not undergo blood tests. In addition, for such a prospective design, probably very high number of pediatric patients should be enrolled to have enough PCC children, if we consider that PCC incidence in children is still unknown and, in any case, much rarer than in adults^10^. Last, our coagulation profile was limited to routinely available studies, while functional or new methodologies to investigate endothelial inflammation and endothelium/platelet hyperactivation/hyperreactivity have not been done, such as assessment of microclots (which have been increasingly studied in adults), thromboelastrography or platelet cytofluorimetry. However, as our study was a pilot preliminary hypothesis, we are convinced that our results now fully support more advanced studies investigating coagulation issues in PCC. Last, we did not enroll a control group of children that never had SARS-CoV-2 infection. However, as it is expected that the large majority of children encountered the virus since the beginning of Omicron wave, and with the growing vaccination rates that make more difficult to understand which children had or not Covid-19, several international experts (including the ISARIC collaborators) are agreeing that a perfect control group is difficult to be obtained and that recovered children may be the best and most realistic control group of PCC children.

In conclusion, our study showed that most children after initial SARS-CoV-2 infection have normal coagulation profile, although the subgroup of PCC children with a more severe spectrum characterized with ≥ 3 persisting symptoms, had a statistically significant higher probability of having mild abnormal D-Dimer levels when compared with children that fully recovered from acute SARS-CoV-2 infection. These findings, along with available literature from adult studies, support the possibility that chronic endothelial inflammation may play a role in pediatric PCC and reinforce the need of performing further studies to better investigate the role of endothelial/platelet hyperactivation and hyperreactivity or chronic endothelial inflammation in pediatric PCC.

## Supplementary Information

Supplementary Information.

## Data Availability

Available upon reasonable request to the corresponding author.
