# Supplementary material for: Extended coagulation profile of children with Long Covid: a prospective study
Source: Sci Rep. 2022 Nov 1;12:18392. doi: 10.1038/s41598-022-23168-y (PMC9626616; doi:10.1038/s41598-022-23168-y)
Supplement: Supplementary file 1 — Supplementary Information. [file 41598_2022_23168_MOESM1_ESM.docx]

**Extended coagulation profile of children with Post Covid Condition (Long Covid) compared with those that fully recovered from SARS-CoV-2 infection: a prospective study**

**Supplementary material**

|  | **Patients not fulfilling PCC≥ 3 definition**  **(n=60)** | **Patients with persistence of more than 3 symptoms (PCC≥ 3)**  **(n=15)** | **p** |
| --- | --- | --- | --- |
| **Age (y)**  median IQR | 10 (4) | 13,2 (6,8) | 0,02 |
| **Number of symptoms during the acute phase**  median IQR | 3 (2) | 5 (5) | 0,008 |
| **Fever, n (%)** | 40 (66,7%) | 12 (80%) | 0,37 |
| **Days of fever during the acute phase**  median IQR | 2 (2) | 3 (5) | 0,01 |
| **Follow-up (months)** median IQR | 3,1 (4,7) | 5,4 (6) |  |
| **Rhinitis, n (%)** | 23 (38,3%) | 4 (26,7%) | 0,55 |
| **Anosmia, n (%)** | 15 (25%) | 7 (46,7%) | 0,09 |
| **Dysgeusia, n (%)** | 13 (21,7%) | 5 (33,3%) | 0,34 |
| **Cough, n (%)** | 19 (31,7%) | 8 (53,3%) | 0,11 |
| **Dyspnea at rest, n (%)** | 2 (3,3%) | 2 (13,3%) | 0,17 |
| **Dyspnea under exertion, n (%)** | 3 (5%) | 2 (13,3%) | 0,26 |
| **Asthma, n (%)** | 1 (1,7%) | 1 (6,7%) | 0,36 |
| **Chest pain, n (%)** | 3 (5%) | 4 (26,7%) | 0,02 |
| **Joint pain, n (%)** | 7 (11,7%) | 5 (33,3%) | 0,04 |
| **Muscle pain, n (%)** | 10 (16,7%) | 9 (60%) | 0,001 |
| **Asthenia, n (%)** | 20 (33,3%) | 8 (53,3%) | 0,15 |
| **Headache, n (%)** | 22 (36,7%) | 9 (60%) | 0,1 |
| **GI disorders, n (%)** | 7 (11,7%) | 3 (20%) | 0,4 |
| **Rash, n (%)** | 4 (6,7%) | 2 (13,3%) | 0,59 |

***Tab.S1*** *Differences in demographics and clinical characteristics during acute SARS-CoV-2 infection between patients with with persistence of 3 or more symptoms at follow-up (PCC*≥3*) vs the rest of the study cohort*

**Abbreviations**: ICU, intensive care unit; GI, gastrointestinal disorders.

|  | **Patients recovered**  **(n=29)** | **PCC12**  **(n=39)** | **p** |
| --- | --- | --- | --- |
| **Age (y)** median IQR | 10,1 (3) | 10 (7) | 0,56 |
| **Number of symptoms during the acute phase**  median IQR | 2 (2) | 4 (4) | 0,02 |
| **Fever, n (%)** | 17 (58,6%) | 31 (79,5%) | 0,06 |
| **Days of fever during the acute phase**  median IQR | 1 (2) | 2 (2) | 0,001 |
| **Follow-up (months)** median IQR | 3,1 (6) | 4,6 (4,3) | 0,43 |
| **Rhinitis, n (%)** | 12 (41,4%) | 13 (33,3%) | 0,5 |
| **Anosmia, n (%)** | 5 (17,2%) | 13 (33,3%) | 0,14 |
| **Dysgeusia, n (%)** | 5 (17,2%) | 10 (25,6%) | 0,4 |
| **Cough, n (%)** | 8 (27,6%) | 18 (46,2%) | 0,12 |
| **Dyspnea at rest, n (%)** | 1 (3,4%) | 3 (7,7%) | 0,63 |
| **Dyspnea under exertion, n (%)** | 1 (3,4%) | 3 (7,7%) | 0,63 |
| **Asthma, n (%)** | 0 (0%) | 2 (5,1%) | 0,5 |
| **Chest pain, n (%)** | 1 (3,4%) | 6 (15,4%) | 0,22 |
| **Joint pain, n (%)** | 1 (3,4%) | 8 (20,5%) | 0,07 |
| **Muscle pain, n (%)** | 2 (6,9%) | 13 (33,3%) | 0,02 |
| **Asthenia, n (%)** | 7 (24,1%) | 15 (38,5%) | 0,21 |
| **Headache, n (%)** | 10 (34,5%) | 15 (38,5%) | 0,74 |
| **GI disorders, n (%)** | 1 (3,4%) | 6 (15,4%) | 0,1 |
| **Rash, n (%)** | 1 (3,4%) | 4 (10,3%) | 0,38 |

***Tab.S2*** *Differences in demographics and clinical characteristics during acute SARS-CoV-2 infection between patients with Persisting Symptoms at 12 weeks or more (PCC12) follow-up since initial infection and patients fully recovered .* *Only patients assessed after 12 weeks were included in these comparisons*

**Abbreviations**: ICU, intensive care unit; GI, gastrointestinal

|  | **Patients with persistence of less than 3 symptoms**  **and follow-up of at least 12 weeks**  **(n=54)** | **Patients with persistence of more than 3 symptoms (PCC≥3)**  **and follow-up of at least 12 weeks**  **(n=14)** | **P** |
| --- | --- | --- | --- |
| **Age (y)** median IQR | 9,8 (4,9) | 13,1 (7,7) | 0,04 |
| **Number of symptoms during the acute phase**  median IQR | 2,5 (2) | 5 (5) | 0,01 |
| **Fever, n (%)** | 36 (66,7%) | 12 (85,7%) | 0,2 |
| **Days of fever during the acute phase**  median IQR | 2 (2) | 3 (5) | 0,003 |
| **Follow-up (months)** median IQR | 3,4 (4,8) | 5,7 (6,6) | 0,21 |
| **Rhinitis, n (%)** | 21 (38,9%) | 4 (28,6%) | 0,55 |
| **Anosmia, n (%)** | 12 (22,2%) | 6 (42,9%) | 0,12 |
| **Dysgeusia, n (%)** | 11 (20,4%) | 4 (28,6%) | 0,49 |
| **Cough, n (%)** | 18 (33,3%) | 8 (57,1%) | 0,1 |
| **Dyspnea at rest, n (%)** | 2 (3,7%) | 2 (14,3%) | 0,18 |
| **Dyspnea under exertion, n (%)** | 2 (3,7%) | 2 (14,3%) | 0,18 |
| **Asthma, n (%)** | 1 (1,9%) | 1(7,1%) | 0,37 |
| **Chest pain, n (%)** | 3 (5,6%) | 4 (28,6%) | 0,03 |
| **Joint pain, n (%)** | 5 (9,3%) | 4 (28,6%) | 0,08 |
| **Muscle pain, n (%)** | 7 (13%) | 8 (57,1%) | 0,0001 |
| **Asthenia, n (%)** | 15 (27,8%) | 7 (50%) | 0,11 |
| **Headache, n (%)** | 17 (31,5%) | 8 (57,1%) | 0,08 |
| **GI disorders, n (%)** | 5 (9,3%) | 2 (14,3%) | 0,63 |
| **Rash, n (%)** | 4 (7,4%) | 1 (7,1%) | 1 |

***Tab.S3*** *Analysis performed excluding children with a follow-up of 8-11 weeks; the table shows the differences in demographics characteristics and in clinical characteristics of the acute infection between patients with persistence of more than 3 symptoms at follow-up and patients with persistence of less than 3 symptoms at ≥12 weeks follow-up*

**Abbreviations**: ICU, intensive care unit; GI, gastrointestinal

|  | **Normal values** | **Patients not fulfilling PCC≥ 3 definition**  **(n=60)** | **Patients with persistence of more than 3 symptoms**  **(PCC≥ 3)**  **(n=15)** | **p** |
| --- | --- | --- | --- | --- |
| **VWFAg,** median IQR |  | 87 (24,8) | 89 (36) | 0,38 |
| **Pathological VWFAg,** n (%) | 70-140% | 14 (23,3%) | 4 (26,7%) | 0,75 |
| **RC,** median IQR |  | 87 (24,8) | 88 (30) | 0,7 |
| **Pathological RC,** n (%) | 70-140% | 10 (16,7%) | 3 (20%) | 0,72 |
| **F VIII,** median IQR |  | 91,5 (29) | 96 (33) | 0,59 |
| **Pathological F VIII,** n (%) | 70-140% | 13 (21,7%) | 2 (13,3%) | 0,72 |
| **PT,** median IQR |  | 11 (0,3) | 11,2 (1) | 0,9 |
| **Pathological PT,** n (%) | 0,9-12 seconds | 1 (1,7%) | 2 (13,3%) | 0,1 |
| **aPTT,** median IQR |  | 33,5 (8,2) | 34 (5,7) | 0,5 |
| **Pathological aPTT,** n (%) | 24-37 seconds | 16 (26,7%) | 3 (20%) | 0,75 |
| **INR,** median IQR |  | 1,1 (0,2) | 1,1 (0,1) | 0,26 |
| **Pathological INR,** n (%) | 0,9-1,2 | 0 | 0 |  |
| **Fibrinogen,** median IQR |  | 239,5 (110) | 298 (73) | 0,06 |
| **Pathological fibrinogen,** n (%) | 200-400 mg/dl | 22 (36,7%) | 3 (20%) | 0,36 |
| **D-dimer,** median IQR |  | 268 (246) | 590 (433) | 0,02 |
| **Pathological d-dimer,** n (%) | <500 ng/ml | 14 (23,3%) | 9 (60%) | 0,006 |

***Tab.S4*** *Differences in the coagulation profile between patients with persistence of 3 or more symptoms at follow-up and patients with persistence of less than 3 symptoms*

**Abbreviations**: VWFAg, Von Willebrand Factor Antigen; RC, ristocetin cofactor; F VIII, Factor VIII; PT, prothrombin time; aPTT, activated partial thromboplastin time.

**Supplementary figure 1** Prevalence of other symptoms at follow-up

**ISARIC PEDIATRIC POST COVID CONDITION SURVEY**

All patients were evaluated during an in-person medical evaluation with a pediatrician collecting the history and clinical examination. Symptoms were reported by the families, as well duration of fever during acute disease. About symptoms, we consider the report of symptoms like “tachycardia/palpitations” reliable as during a clinical examination at rest heart rate may be normal, but only specific circumstances lead to inappropriate changes in heart rate, asa widely reported by literature and family associations. Importantly, we are also performing a study measuring 24hours ECG with heart rate variability in children with Long Covid, whose results are not yet available. Similarly, all Long Covid children undergo Cardiopulmonary Excersice testing, this is another ongoing project with exciting preliminary results.

Regarding minor symptoms like runny nose or ocular hyperemia, we don’t consider them as long covid if they do not have a negative impact on daily life. We are very rigorous on our classification. About rash, the same except (like happened in one case) there is an unexplained chronic urticaria with angioedema which required multiple ED evaluations and chronic use of medications.

**FOR CHILDREN AND YOUNG PEOPLE**

**(less than 18 years of age)**

| PLEASE MARK YOUR INITIALS AGAINST EACH STATEMENT WITH WHICH YOU AGREE: | *Add your Initials or tick the box:* | |
| --- | --- | --- |
| I give my consent for the information I provide in this study to be used as advised. |  |  |
| I would like to continue to be sent this survey via email, post or to be contacted via telephone follow up every 3 to 6 months for a maximum of 3 years after my respiratory illness,.  If yes, please enter your contact details here:  E-mail:  Mobile phone number:  Home telephone number: | YES | NO |
|  |  |  |
| Please enter your details  Your first name: Surname: _______________________  Town/City of residence: Postcode: _______________  Your signature: ________________________  If you are completing the survey on behalf of your child please also enter your details below:  Your first name: Surname:  Your signature: _____________________________ | | |

Local hospital ID:

| **1.** **About you (if the survey is completed by an adult or carer, all questions relates to the child)** |
| --- |
| **Sex/Gender:** ❑ Male ❑ Female ❑ Non-binary ❑ Prefer not to say |

| **2.** **About your Covid-19 or for control group other respiratory infection**  (if the survey is completed by an adult or carer, all questions relates to the child) |
| --- |
| **Date you completed this survey (DD/MM/YYYY):** [_D_][_D_]/[_M_][_M_]/[_2_][_0_][_2_][_Y_]  **What is your date of birth (DD/MM/YYYY):** [_D_][_D_]/[_M_][_M_]/[_2_][_0_][_Y_][_Y_] |
| **2a. How were you diagnosed with Covid-19?**  **** Laboratory confirmed (positive test)  Physician/doctor confirmed  **Estimated date of your most recent positive SARS-CoV-2 /Covid-19 test:**  [_D_][_D_]/[_M_][_M_]/[_2_][_0_][_2_][_Y_] ❑ Not applicable |
| **2b. What symptoms did you experience at onset of Covid-19 (cases), OR onset of other respiratory infection (controls)?** |
| **What symptoms did you experience in the first 14 days of your illness?**  (tick all that you experienced when you first became unwell) 🞏Fever ≥ 38^o^C 🞏Runny nose 🞏 Headache  🞏Sore throat 🞏Muscle pain 🞏Abdominal pain 🞏Vomiting 🞏Diarrhoea  🞏 Cough 🞏Shortness of breath 🞏 Fatigue 🞏 Pain on breathing 🞏Chest pain 🞏Loss or disturbed smell  🞏Loss or disturbed taste 🞏Confusion 🞏Brain fog* 🞏Other symptoms:________________  🞏No symptoms  *****Brain fog (often described as a feeling ‘foggy’, confusion, short term memory problems, indecisive, not being able to think clearly) |
| **3. Hospitalisation** |
| **Have you been admitted to hospital due to this illness (Covid-19)?** ❑ Yes ❑ No If yes complete the below, if no skip to question 4.  **Roughly at what date were you first admitted to hospital?** [_D_][_D_]/[_M_][_M_]/[_2_][_0_][_2_][_Y_]  **Roughly at what date were you first discharged from hospital?** [_D_][_D_]/[_M_][_M_]/[_2_][_0_][_2_][_Y_]  **Did you spend any time in an Intensive Care Unit (P/ICU)?** ❑ Yes ❑ No ❑ Not sure  **Did your receive oxygen** **(e.g. via a mask, or nose cannula)?** ❑ Yes ❑ No ❑ Not sure  **Have you been re-admitted to hospital after the first acute illness?** ❑ Yes ❑ No  **If yes, how many times**: [_Number_] please specify main reason/reasons:___________________________ |
| **4. About your state of health before you were diagnosed with Covid-19 or :** |
| \| **Have you been physicians diagnosed or received treatment/support for any of the following prior to onset of your illness that are still ongoing? (answer with a tick in the box)** \| \| \| \| \| \| \| --- \| --- \| --- \| --- \| --- \| --- \| \|  \| **Yes** \| **No** \|  \| **Yes** \| **No** \| \| \| Neurological/Neuro-disability \|  \|  \| Immune system diseases \|  \|  \| \| \| Gut/stomach problems \|  \|  \| Genetic conditions \|  \|  \| \| \| Heart diseases \|  \|  \| Diabetes (if yes indicate type: □ Type 1 □ Type 2) \|  \|  \| \| \| Respiratory diseases (not including asthma) \|  \|  \| Other endocrine illness (not diabetes) \|  \|  \| \| \| Asthma (doctors diagnosed) \|  \|  \| Renal/Kidney problems \|  \|  \| \| \| Allergic rhinitis/hay fever \|  \|  \| Excessive weight or obesity \|  \|  \| \| \| Food allergy \|  \|  \| Malnutrition \|  \|  \| \| \| Atopic dermatitis/Eczema \|  \|  \| Depression \|  \|  \| \| \| Rheumatology  *(e.g. arthritis, or inflammation of the joints)* \|  \|  \| Anxiety \|  \|  \| \| \| Sickle cell disease \|  \|  \| HIV \|  \|  \| \| \| Haematology *(other blood diseases)* \|  \|  \| TB (tuberculosis) \|  \|  \| \| \| Oncology (cancer, including lymphoma) \|  \|  \| Other (please indicate): \|  \|  \| \|  \|   **Were you born prematurely (<37 weeks)?** ❑ Yes ❑ No ❑ Not sure  **Have you ever sought support from a child /adolescent mental health/psychological services before the Covid-19 pandemic?** ❑ Yes ❑ No  **Prior to Covid-19 or other respiratory illness onset, how was your physical health in general?**  ❑ Very poor ❑ Poor ❑ Ok ❑ Good ❑ Very good  **Prior to Covid-19 or other respiratory illness onset, how would you describe your mental /psychological health in general?** ❑ Very poor ❑ Poor ❑ Ok ❑ Good ❑ Very good |
| **5.** Are you experiencing any of these symptoms**,** which **were NOT present** before start of your Covid-19 / other respiratory illness (control group)? (Indicate if you have a symptom (tick Yes) or if you do not have a specific symptom (tick no)) |
| \| **Symptoms** \| **Tick Yes/No** \| \| --- \| --- \| \| Nasal congestion / rhinorrhea \| ❑ Yes ❑ No \| \| Difficulty breathing /chest tightness \| ❑ Yes ❑ No \| \| Chest pain \| ❑ Yes ❑ No \| \| Persistent cough \| ❑ Yes ❑ No \| \| Problems with balance \| ❑ Yes ❑ No \| \| Persistent muscle pain \| ❑ Yes ❑ No \| \| Joint pain or swelling \| ❑ Yes ❑ No \| \| Headache \| ❑ Yes ❑ No \| \| Dizziness/ light headedness \| ❑ Yes ❑ No \| \| Problems seeing/blurred vision \| ❑ Yes ❑ No \| \| Disturbed smell/Loss of smell \| ❑ Yes ❑ No \| \| Disturbed taste/Loss of taste \| ❑ Yes ❑ No \| \| Insomnia *(hard to fall asleep, hard to stay asleep)* \| ❑ Yes ❑ No \| \| Hypersomnia *(excessive daytime sleepiness or prolonged nighttime sleep)* \| ❑ Yes ❑ No \| \| Tingling feeling/”pins and needles” \| ❑ Yes ❑ No \| \| Fainting/black outs \| ❑ Yes ❑ No \| \| Confusion/loss of concentration \| ❑ Yes ❑ No \| \| Fatigue \| ❑ Yes ❑ No \| \| Poor appetite \| ❑ Yes ❑ No \| \| Diarrhea \| ❑ Yes ❑ No \| \| Stomach/ abdominal pain \| ❑ Yes ❑ No \| \| Feeling nauseous/persistent vomiting \| ❑ Yes ❑ No \| \| Constipation \| ❑ Yes ❑ No \| \| Palpitations (heart racing) \| ❑ Yes ❑ No \| \| Variations in heart rate (tachycardia or bradycardia) \| ❑ Yes ❑ No \| \| Skin rash \| ❑ Yes ❑ No \| \| Other New Symptoms, if yes, specify: \| \| \|  \| \| \|  \| \| |

| **6. Please let us know of any additional comments about your respiratory illness (Covid-19 or other declared):** |
| --- |
|  |
| **End of survey** |
| **Thank you for your time!** |
